# Blood Pressure Load: An Effective Indicator of Systemic Circulation Status in Individuals With Acute Altitude Sickness

**DOI:** 10.3389/fcvm.2021.765422

**Published:** 2022-01-03

**Authors:** Renzheng Chen, Xiaowei Ye, Mengjia Sun, Jie Yang, Jihang Zhang, Xubin Gao, Chuan Liu, Jingbin Ke, Chunyan He, Fangzhengyuan Yuan, Hailin Lv, Yuanqi Yang, Ran Cheng, Hu Tan, Lan Huang

**Affiliations:** ^1^Institute of Cardiovascular Diseases of Chinese People's Liberation Army (PLA), The Second Affiliated Hospital, Third Military Medical University (Army Medical University), Chongqing, China; ^2^Department of Cardiology, The Second Affiliated Hospital, Third Military Medical University (Army Medical University), Chongqing, China

**Keywords:** ambulatory blood pressure monitoring, blood pressure load, area under the blood pressure curve, acute mountain sickness, high altitude

## Abstract

**Background:** Acute high altitude (HA) exposure results in blood pressure (BP) variations in most subjects. Previous studies have demonstrated that higher BP is potentially correlated with acute mountain sickness (AMS). The BP load may be of clinical significance regarding systemic circulation status.

**Objectives:** This study aimed to examine HA-induced BP changes in patients with AMS compared to those in healthy subjects. Further, we provided clinical information about the relationship between variations in 24-h ambulatory parameters (BP level, BP variability, and BP load) and AMS.

**Methods:** Sixty-nine subjects were enrolled and all participants ascended Litang (4,100 m above sea level). They were monitored using a 24-h ambulatory blood pressure device and underwent echocardiography within 24 h of altitude exposure. The 2018 Lake Louise questionnaire was used to evaluate AMS.

**Results:** The AMS group comprised more women than men [15 (65.2%) vs. 13 (28.3%), *P* < 0.001] and fewer smokers [4 (17.4%) vs. 23 (50.0%), *P* = 0.009]. The AMS group exhibited significant increases in 24-h BP compared to the non-AMS group (24-h SBP variation: 10.52 ± 6.48 vs. 6.03 ± 9.27 mmHg, *P* = 0.041; 24-h DBP variation: 8.70 ± 4.57 vs. 5.03 ± 4.98 mmHg, *P* = 0.004). The variation of mean 24-h cBPL (cumulative BP load) (mean 24-h cSBPL: 10.58 ± 10.99 vs. 4.02 ± 10.58, *P* = 0.016; 24-h mean cDBPL: 6.03 ± 5.87 vs. 2.89 ± 4.99, *P* = 0.034) was also obviously higher in AMS subjects than in non-AMS subjects after HA exposure. 24-h mean cSBPL variation (OR = 1.07, *P* = 0.024) and 24-h mean cDBPL variation (OR = 1.14, *P* = 0.034) were independent risk factors of AMS. Moreover, variation of 24-h mean cSBPL showed a good correlation with AMS score (*R* = 0.504, *P* < 0.001).

**Conclusions:** Our study demonstrated that patients with AMS had higher BP and BP load changes after altitude exposure than healthy subjects. Excessive BP load variations were associated with AMS. Thus, BP load could be an effective indicator regarding systemic circulation status of AMS.

## Introduction

An increasing number of people are exposed to high altitudes (HA) for various reasons. However, HA can induce a series of cardiovascular responses due to hypobaric and hypoxic influences (1, 2). The changes in BP are particularly prominent. Some maladaptive individuals may suffer acute mountain sickness (AMS), which is characterized by headache and other related symptoms (3, 4). Previous studies have indicated that certain cardiovascular system indicators including electrocardiogram changes and cardiac systolic function are related to the occurrence and development of AMS (5, 6). Moreover, excessive elevation of arterial BP is detrimental to the body and is considered to be closely associated with AMS (7). Therefore, accurate and effective monitoring of the BP after HA exposure is important for AMS prevention and treatment.

The BP load mainly refers to the degree of arterial BP above set thresholds (8). Although studies have demonstrated that the BP load is an important predictor of adverse cardiovascular events (9), the traditional method of BP load calculation (BP readings above normal) is of limited clinical significance for risk predictions based on the 24-h BP level (10). The BP load calculated by the area under the time-pressure curve effectively reflects for the BP status (11). During acute HA exposure, adverse cardiovascular responses are primarily related to sympathetic hyperactivation (12). The BP load indicates the fluctuation in the BP caused by sympathetic activity and is better calculated in this way compared to the traditional method. Therefore, we propose that it may be a significant indicator of the BP status in AMS with superior predictive value.

Thus, in the present study, we obtained 24-h ambulatory BP (ABP) at both low altitude (LA) and HA in patients with AMS as well as in non-AMS susceptible subjects. Two different methods are used to calculate the BP load. We sought to ascertain whether there was an association between the BP load variations at HA in individuals with AMS and aimed to provide related information.

## Materials and Methods

### Study Population and Ethical Considerations

This prospective observational cohort study involving 24-h ABP monitoring was conducted in Chengdu, China, in 2019. All subjects underwent a comprehensive medical examination before embarking on the expedition at LA (Chongzhou, 400 m above sea level) and underwent ABP monitoring and echocardiography both at the LA and HA (Litang, 4100 m above sea level). The exclusion criteria were as follows: (1) Any clinical conditions that may affect HA-adaptation, including known pulmonary diseases, cardiovascular diseases, hematological diseases, and so on. (2) Use of any medication. (3) Long term residence history and recent exposure history of altitude (last 6 months). Notably, subjects diagnosed with hypertension first according to the ABP data at LA (24-h BP > 130/80 mmHg and/or daytime BP > 135/85 mmHg and/or nighttime BP > 120/70 mmHg) (13) and 24-h ABP monitor recordings of <80% of the total data recorded (14) were excluded. Finally, 69 subjects were included, all of whom provided written informed consent. This study was performed in agreement with the Declaration of Helsinki and was approved by the Human Ethics Committee of the Xinqiao Hospital, Third Military Medical University (Identification code, 201907501) which registered at www.chictr.org.cn (ChiCTR-TRC-No.1900025728).

### BP Measurement and BP Load Calculation

**Figure 1:**
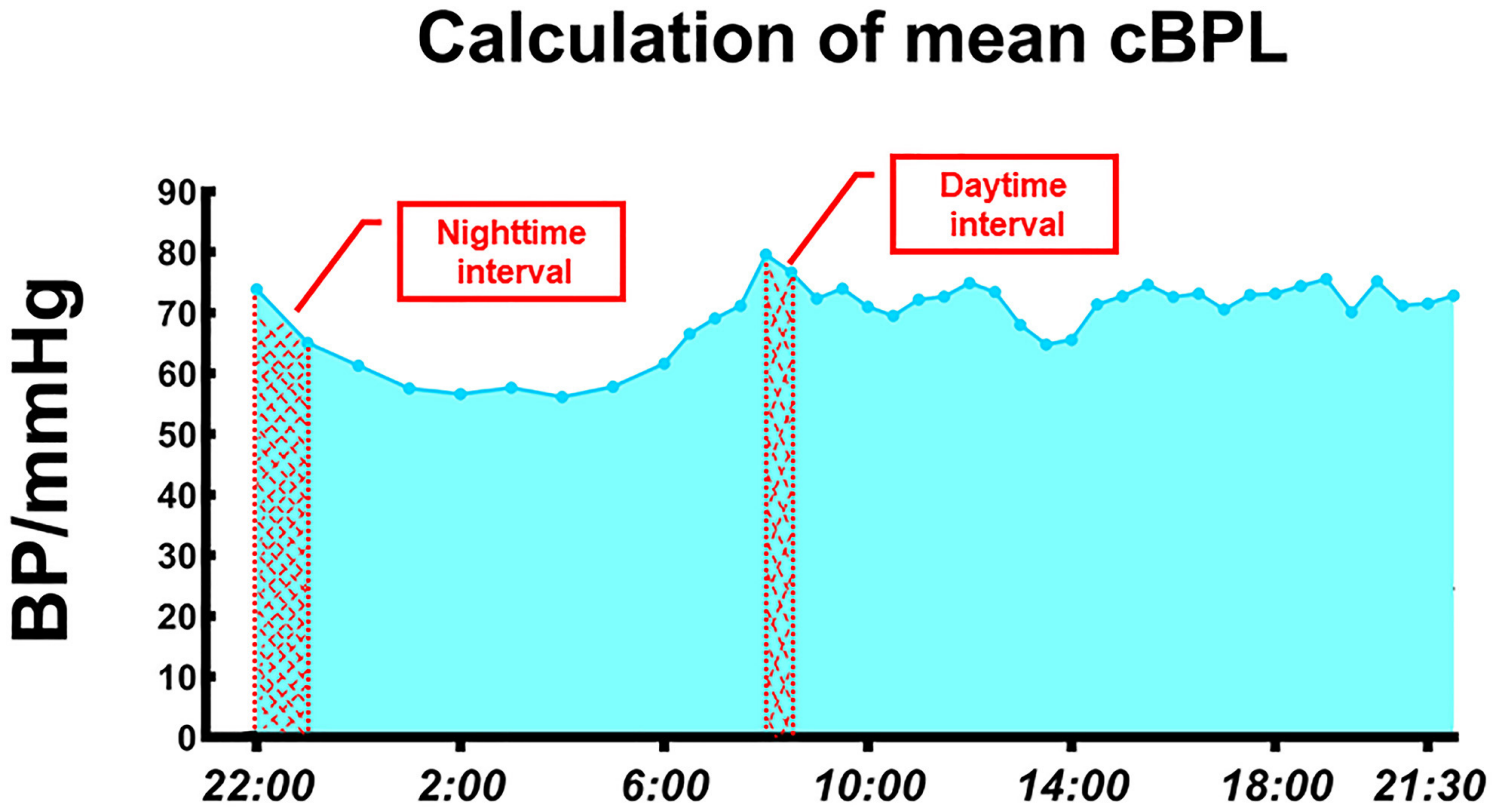
Calculation of mean cBP load. BP, blood pressure; cBPL, cumulative blood pressure load.

Two well-trained cardiovascular physicians performed ABP measurements using an ABM device (Spacelabs 90207, Redmond, WA, USA). The BP cuff was applied to the non-dominant arm on a weekday morning and was removed 24 h later. All participants were asked to remain still during the measurements. The subjects were instructed to avoid any unusual physical activities and follow a standard schedule at both LA and HA. Daytime and nighttime were defined as 6:00 to 22:00 and 22:00 to 6:00, respectively (11). The BP was recorded every 30 min during the daytime and every 60 min at night (15). The average real variability (ARV) of SBP and DBP was calculated as described in a previous study (16). BP load is the proportion of times that the BP exceeds normal values of the total number of recorded BP measurements during a certain period of time (day, night, and over 24 h) (17). To more accurately reflect the pressure load of the blood vessels, we calculated the cumulative BP load (cBPL), which is defined as the area between the fluctuating ABP curve and the time axis (11). Fitting the fluctuating BP curve by connecting adjacent data points with straight lines was used to determine the magnitude and durations of 24-h cumulative BP increases. By deconstructing the curve into many small trapezoids, we determine their areas, and summed the values. Due to missing data for some periods, we calculated the mean cBPL by dividing the total area by the number of periods measured. The interval between BP measurements at night (1 h) is twice as long as that during the day (30 min) (Figure 1); therefore, the number of periods measured doubled in the calculation of mean cBPL.

### Echocardiography Examination

Echocardiographic examination was performed using an ultrasound machine (CX50, Philips Ultrasound System, Andover, MA, USA) to acquire left ventricular (LV) data. Images were saved digitally for subsequent offline analysis using QLAB software (QLAB 10.5, Philips Healthcare, Andover, MA, USA). We measured the LV dimensions and volumes by a computerized analysis software system. Ejection fraction (EF) was calculated by the LV volume data. Mitral inflow pattern from the tips level was analyzed for peak early diastolic velocity (E) as well as late diastolic velocity (A), and E/A. Reproducibility of main echocardiographic measurements was assessed in 20 randomly selected subjects. Interobserver variability was tested by two different physicians, and intraobserver variability was tested by the same physician at least 1 month apart. Both the interobserver and intraobserver variabilities were determined using the intraclass correlation coefficient (ICC). The ICC values were all over 0.85 and *p*-value < 0.001.

### Assessment of AMS

**Figure 2:**
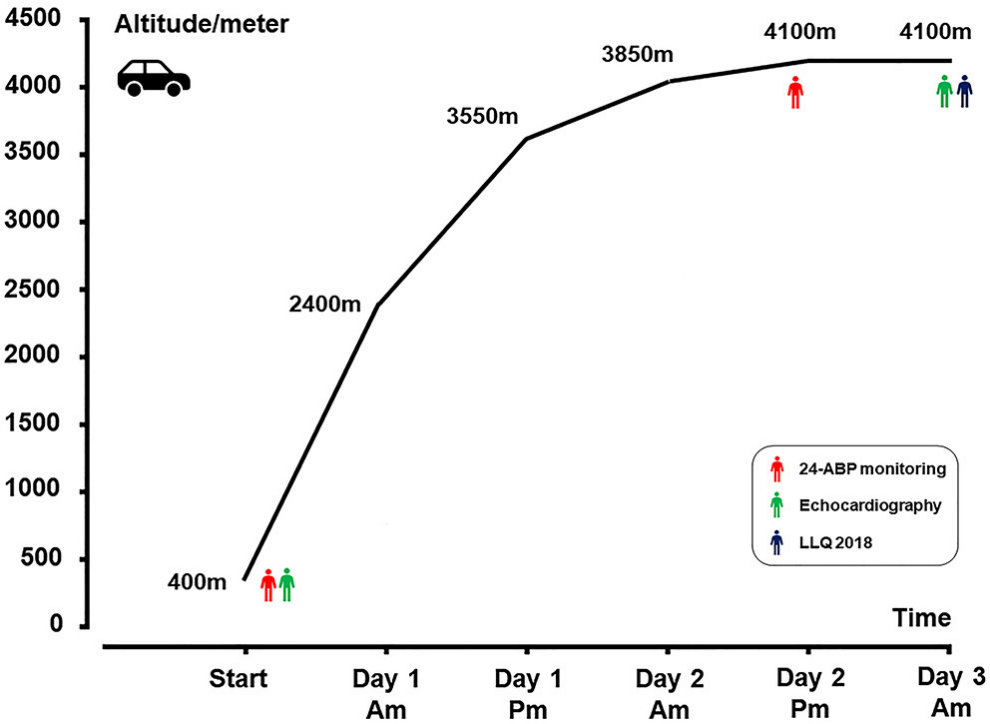
Ascent profile. ABP, ambulatory blood pressure monitoring; LLQ, Lake Louise questionnaire.

All subjects traveled by automobiles from LA to HA within 2 days (Figure 2). AMS was diagnosed using the latest Lake Louise questionnaire (2018) ~8 h after arriving at HA. This comprises a four-item self-administered questionnaire based on the most frequent AMS symptoms: headache, dizziness, light-headedness, gastrointestinal symptoms, and fatigue. Participants completed the questionnaire with the assistance of an experienced physician. Each item is scored from 0 to 3, according to the severity of the symptom (0: no symptoms, 1: mild symptoms, 2: moderate symptoms; and 3: severe symptoms). AMS was defined as a total score ≥ 3, with at least one point from the headache (18).

### Statistical Analysis

Continuous variables are presented as means ± standard deviations. Differences in measurements between men and women with normal distribution were tested using an independent-sample *t*-test, whereas the data that did not fit a normal distribution were analyzed using the Mann–Whitney U test. Categorical data were presented as percentages (%) and were compared using the chi-square test, continuity correction, or Fisher's exact test, as appropriate. Binary logistic regression was used to predict the risk factors of AMS. Spearman correlation coefficients were used to determine the correlation between the different BP index variations after acute HA exposure and AMS scores. Statistical significance was set at *P* < 0.05. Statistical analyses were performed using SPSS software Version 26 (IBM, Armonk, NY, USA). Statistical power calculations were performed using the PASS software, version 11 (NCSS, LLC, Kaysville, UT, USA). The results suggested that 69 subjects would provide more than 75% power to detect differences in targeted BP parameters between subgroups using a two-sided alpha of 0.05.

## Results

### Demography and AMS-Related Symptoms Parameters

Twenty-three subjects developed AMS in the final data analysis. Age and BMI did not differ significantly between the two groups. The AMS group comprised a higher proportion of women (65.2 vs. 28.3%, *P* = 0.001) but a lower proportion of smokers (17.4 vs. 50.0%, *P* = 0.009). The AMS score and the percentage of AMS-related symptoms were also significantly higher in the AMS group (Table 1).

**Table 1:** Demographic and AMS symptoms parameters.

**Variables**	**Al** **(***n*** = 69)**	**AMS** **(***n*** = 23)**	**Non-AMS** **(***n*** = 46)**	***P*-value**
Age, years	27.10 ± 7.88	27.57 ± 7.06	26.87 ± 8.33	0.718
BMI, kg/m^2^	21.70 ± 2.05	21.53 ± 2.28	21.78 ± 1.95	0.631
Females	28 (40.6%)	15 (65.2%)	13 (28.3%)	<0.001
Tibetan	1 (1.4%)	0 (0.0%)	1 (2.2%)	1.000
Alcohol	14 (20.3%)	4 (17.4%)	10 (21.7%)	1.000
Cigarette smoking	27 (39.1%)	4 (17.4%)	23 (50.0%)	0.009
AMS score	2.42 ± 1.77	4.48 ± 1.31	1.39 ± 0.80	<0.001
Headache	52 (75.4%)	22 (95.7%)	30 (65.2%)	0.006
Dizziness	37 (53.6%)	21 (91.3%)	16 (34.8%)	<0.001
Gastrointestinal symptoms	13 (18.8%)	11 (47.8%)	2 (4.3%)	<0.001
Fatigue	37 (53.6%)	23 (100.0%)	14 (30.4%)	<0.001

*Values are presented as mean ± standard deviation and percentage (%)*.

*BMI, body mass index; AMS, acute mountain sickness*.

### Baseline Parameters

Patients with AMS had a higher nighttime heart rate (HR) (62.71 ± 8.29 vs. 57.58 ± 7.71 beat/min, *P* = 0.017) and a lower daytime BP (daytime SBP, 114.94 ± 10.77 vs. 122.60 ± 6.50 mmHg, *P* = 0.004; daytime DBP, 69.51 ± 5.83 vs. 72.58 ± 4.55 mmHg, *P* = 0.019) at LA. Both 24-h SBP load and daytime SBP load were lower in the AMS group than in the non-AMS group at LA (24-h SBP load: 15.75 ± 9.53 vs. 21.63 ± 10.25%, *P* = 0.025; daytime SBP load: 15.67 ± 11.35 vs. 23.09 ± 11.45%, *P* = 0.013). Moreover, 24-h mean cSBPL (92.81 ± 10.36 vs. 98.47 ± 7.86, *P* = 0.014), daytime mean cSBPL (114.98 ± 11.34 vs. 122.70 ± 57.30, *P* = 0.001), and daytime mean cDBPL (69.15 ± 6.02 vs. 72.75 ± 4.64, *P* = 0.008), which were calculated using the BP-time curve area, were higher in non-AMS participants at LA. Besides, there was no obvious difference in the ARV and cardiac function between the two groups at LA.

### Parameters After Acute HA Exposure

**Figure 3:**
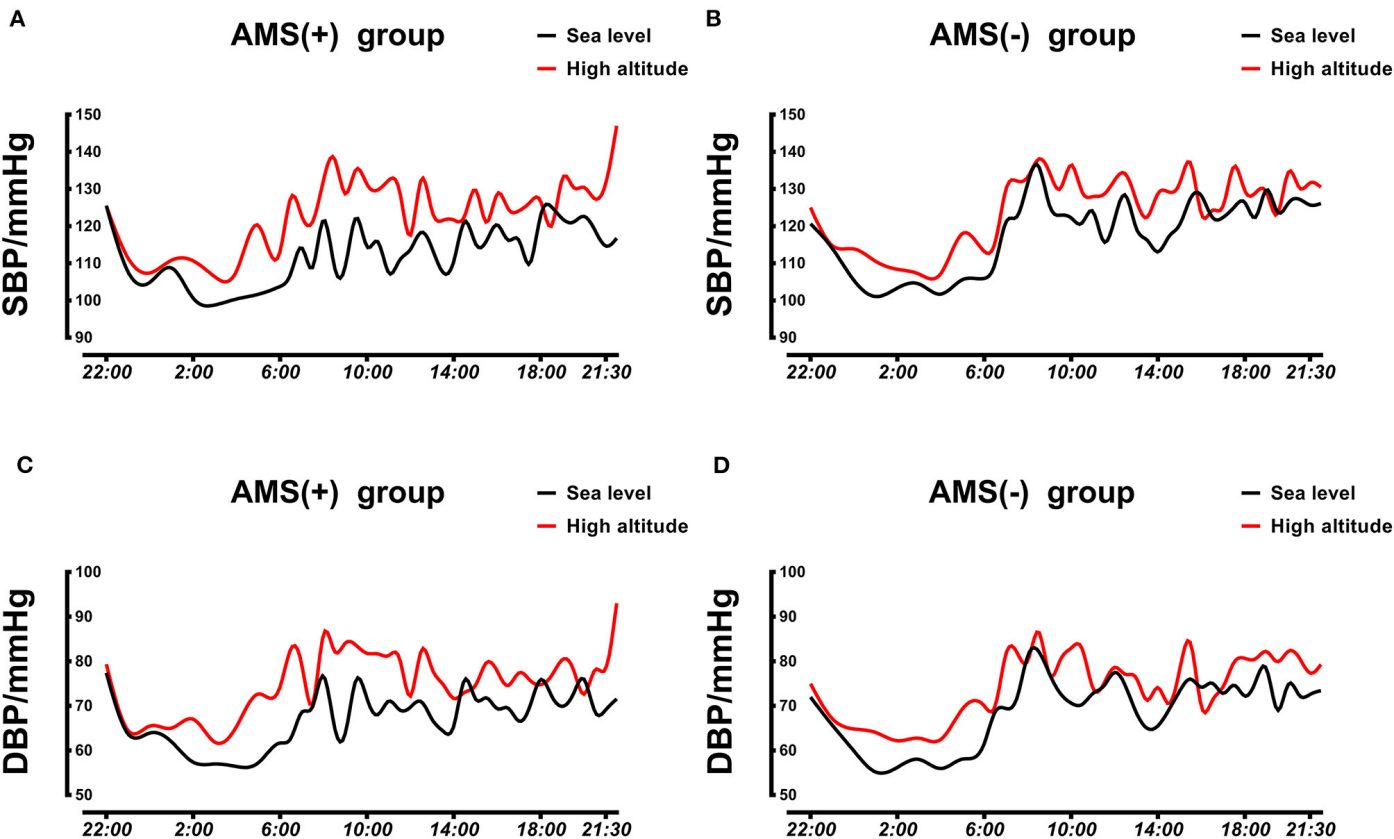
Averaged 24-h SBP and DBP profiles in AMS and non-AMS subjects. **(A)** Averaged 24-h SBP in AMS subjects at LA and HA. **(B)** Averaged 24-h SBP in non-AMS subjects at LA and HA. **(C)** Averaged 24-h DBP in AMS subjects at LA and HA. **(D)** Averaged 24-h DBP in non-AMS subjects at LA and HA. SBP, systolic blood pressure; DBP, diastolic blood pressure; LA, low altitude; HA, high altitude; AMS, acute mountain sickness.

After arriving at HA, AMS subjects showed a lower SpO2 at HL, but no statistical difference was found in SpO2 variation between the two groups. Both daytime and nighttime BP increased after acute HA exposure in each group (Figure 3). 24-h BP increased significantly in the AMS group compared to the non-AMS group (24-h SBP variation: 10.52 ± 6.48 vs. 6.03 ± 9.27 mmHg, *P* = 0.041; 24-h DBP variation: 8.70 ± 4.57 vs. 5.03 ± 4.98, *P* = 0.004) which attributed to a higher elevation of daytime BP in subject with AMS (daytime SBP variation: 12.68 ± 8.91 vs. 6.46 ± 11.12, *P* = 0.023; daytime DBP variation: 9.15 ± 5.41 vs. 5.05 ± 6.35 mmHg, *P* = 0.010) (Table 2). 24-h DBP load variation (19.98 ± 11.42 vs. 11.21 ± 5.41%, *P* = 0.011) and daytime DBP load variation (19.71 ± 10.30 vs. 10.81 ± 6.66%, *P* = 0.008) were significantly higher in the AMS group compared to the non-AMS group. Furthermore, 24-h mean cBPL variation (24-h mean cSBPL 10.58 ± 10.99 vs. 4.02 ± 10.58, *P* = 0.016; 24-h mean cDBPL: 6.03 ± 5.87 vs. 2.89 ± 4.99, *P* = 0.034) and daytime mean cBPL variation (daytime mean cSBPL: 15.55 ± 13.99 vs. 6.77 ± 11.59, *P* = 0.007; daytime mean cDBPL: 9.00 ± 5.66 vs. 4.63 ± 6.97, *P* = 0.011) were also obviously higher in AMS subjects than in non-AMS subjects (Table 2). In addition, there was no obvious difference in the ARV and cardiac function between the two groups (Table 2).

**Table 2:** Effect of acute HA exposure.

**Variables**	**LA**			**HA**			**Δ** **HA-LA**		
	**AMS** **(***n*** = 23)**	**Non-AMS** **(***n*** = 46)**	***P*** **-value**	**AMS** **(***n*** = 23)**	**Non-AMS** **(***n*** = 46)**	***P*** **-value**	**AMS** **(***n*** = 23)**	**Non-AMS** **(***n*** = 46)**	***P*** **-value**
SpO_2_, %	96.74 ± 1.76	97.28 ± 1.38	0.315	86.00 ± 2.39	87.37 ± 2.98	0.039	−10.74 ± 3.14	−9.91 ± 3.17	0.229
24-h HR, bpm	73.99 ± 8.60	73.10 ± 6.15	0.620	84.90 ± 7.21	83.01 ± 7.78	0.335	10.90 ± 8.33	9.92 ± 8.15	0.640
Daytime HR, bpm	77.07 ± 9.84	77.43 ± 6.98	0.862	87.47 ± 6.55	87.24 ± 8.16	0.907	10.39 ± 8.69	9.80 ± 8.48	0.788
Nighttime HR, bpm	62.71 ± 8.29	57.58 ± 7.71	0.017	72.64 ± 11.59	68.12 ± 9.20	0.083	9.93 ± 13.24	10.54 ± 9.68	0.827
**BP characteristic, mmHg**									
24-h SBP	113.06 ± 9.29	119.43 ± 6.28	0.006	123.58 ± 8.43	125.46 ± 8.90	0.404	10.52 ± 6.48	6.03 ± 9.27	0.041
Daytime SBP	114.94 ± 10.77	122.60 ± 6.50	0.004	127.62 ± 8.09	129.06 ± 10.11	0.557	12.68 ± 8.91	6.46 ± 11.12	0.023
Nighttime SBP	105.92 ± 8.53	107.14 ± 9.57	0.607	112.28 ± 12.52	112.73 ± 8.21	0.859	6.36 ± 9.14	5.59 ± 10.4	0.763
24-h DBP	67.84 ± 5.17	69.94 ± 4.31	0.079	76.54 ± 5.59	74.97 ± 4.73	0.226	8.70 ± 4.57	5.03 ± 4.98	0.004
Daytime DBP	69.51 ± 5.83	72.58 ± 4.55	0.019	78.66 ± 5.34	77.63 ± 5.33	0.450	9.15 ± 5.41	5.05 ± 6.35	0.010
Nighttime DBP	61.68 ± 6.51	59.98 ± 5.97	0.208	67.32 ± 9.83	65.59 ± 6.89	0.454	5.65 ± 8.84	5.62 ± 7.10	0.987
**BP load characteristic**									
24-h SBP load, %	15.75 ± 9.53	21.63 ± 10.25	0.025	35.84 ± 21.18	33.88 ± 16.46	0.674	20.08 ± 14.22	12.25 ± 18.15	0.075
Daytime SBP load, %	15.67 ± 11.35	23.09 ± 11.45	0.013	36.04 ± 19.64	35.89 ± 17.98	0.960	20.38 ± 14.88	12.71 ± 21.13	0.125
Nighttime SBP load, %	15.66 ± 19.09	16.99 ± 17.39	0.558	34.68 ± 34.00	26.98 ± 20.89	0.792	19.02 ± 27.61	9.99 ± 23.67	0.423
24-h DBP load, %	14.08 ± 11.65	16.63 ± 9.73	0.175	34.06 ± 19.22	27.83 ± 14.43	0.136	19.98 ± 11.42	11.21 ± 5.41	0.011
Daytime DBP load, %	12.91 ± 12.82	15.94 ± 9.60	0.274	32.62 ± 16.64	26.74 ± 14.28	0.132	19.71 ± 10.30	10.81 ± 6.66	0.008
Nighttime DBP load, %	18.37 ± 15.70	19.53 ± 18.32	0.938	39.44 ± 33.93	31.68 ± 26.61	0.484	21.07 ± 28.93	12.15 ± 28.86	0.264
24-h mean cSBPL	92.81 ± 10.36	98.47 ± 7.86	0.014	103.39 ± 11.45	102.49 ± 8.66	0.656	10.58 ± 10.99	4.02 ± 10.58	0.016
Daytime mean cSBPL	114.98 ± 11.34	122.70 ± 57.30	0.001	130.53 ± 14.79	129.511 ± 10.56	0.794	15.55 ± 13.99	6.77 ± 11.59	0.007
Nighttime mean cSBPL	52.35 ± 4.26	53.19 ± 5.11	0.500	56.12 ± 8.06	55.68 ± 3.89	0.810	3.77 ± 6.41	2.50 ± 5.62	0.424
24-h mean cDBPL	55.54 ± 5.45	57.54 ± 4.46	0.085	61.43 ± 5.30	60.43 ± 4.21	0.397	6.03 ± 5.87	2.89 ± 4.99	0.034
Daytime mean cDBPL	69.15 ± 6.02	72.75 ± 4.64	0.008	78.15 ± 5.63	77.38 ± 5.86	0.849	9.00 ± 5.66	4.63 ± 6.97	0.011
Nighttime mean cDBPL	30.33 ± 3.28	29.40 ± 3.23	0.163	32.79 ± 5.54	31.73 ± 3.82	0.420	2.45 ± 5.03	2.33 ± 4.23	0.918
**ARV characteristic, mmHg**									
24-h ARVs	15.60 ± 5.07	17.71 ± 4.58	0.086	19.60 ± 4.84	19.43 ± 5.23	0.901	4.00 ± 4.99	1.72 ± 6.29	0.136
Daytime ARVs	16.57 ± 6.11	18.77 ± 5.32	0.128	21.43 ± 6.09	21.16 ± 5.89	0.860	4.86 ± 5.76	2.39 ± 7.54	0.172
Nighttime ARVs	12.00 ± 6.17	14.40 ± 7.34	0.183	14.01 ± 5.08	13.74 ± 7.09	0.376	2.00 ± 8.71	−0.66 ± 10.01	0.260
24-h ARVd	12.06 ± 4.45	13.59 ± 4.86	0.401	15.40 ± 4.84	14.40 ± 4.36	0.391	3.34 ± 5.38	0.81 ± 5.73	0.078
Daytime ARVd	12.85 ± 5.06	14.54 ± 5.31	0.204	16.75 ± 6.20	15.39 ± 5.25	0.344	3.90 ± 6.28	0.85 ± 7.02	0.083
Nighttime ARVd	8.95 ± 5.09	10.89 ± 7.70	0.215	11.31 ± 3.99	10.52 ± 4.98	0.214	2.36 ± 7.31	−0.37 ± 8.80	0.204
**Cardiac function characteristic**									
EF, %	59.92 ± 4.24	59.11 ± 3.43	0.396	60.68 ± 6.07	61.68 ± 5.21	0.479	0.76 ± 6.37	2.57 ± 5.61	0.231
E/A	1.96 ± 0.71	1.88 ± 0.52	0.698	1.37 ± 0.33	1.46 ± 0.34	0.267	−0.60 ± 0.68	−0.42 ± 0.54	0.311

*Values are presented as mean ± standard deviation*.

*HA, high altitude; LA, low altitude; AMS, acute mountain sickness; HR, heart rate; BP, blood pressure; SBP, systolic blood pressure; DBP, diastolic blood pressure; cSBPL, cumulative systolic blood pressure load; cDBPL, cumulative diastolic blood pressure load; ARV, average real variability; ARVs, average real variability of SBP; ARVd, average real variability of DBP; EF, ejection fraction; E/A, peak early diastolic velocity/late diastolic velocity; Δ, variation after HA exposure*.

### Risk Factors of AMS Associated With BP Variation

We looked for the risk factors of AMS which associated with the BP changes. After adjusting for age, sex, BMI, and smoking status, the results of the regression analysis showed that 24-h SBP variation (OR = 1.08, *P* = 0.036), 24-h DBP variation (OR = 1.21, *P* = 0.007), 24-h SBP load variation (OR = 1.05, *P* = 0.010), 24-h DBP load variation (OR = 1.06, *P* = 0.016), 24-h mean cSBPL variation (OR = 1.07, *P* = 0.024), 24-h mean cDBPL variation (OR = 1.14, *P* = 0.034) were independent risk factors of AMS (Table 3).

**Table 3:** Risk factors of AMS.

**Variable**	**Unadjusted analysis**		**Adjusted analysis**	
	**OR (95% CI)**	***P*** **-value**	**OR (95% CI)**	***P*** **-value**
Δ24-h SBP	1.07 (1.00–1.14)	0.048	1.08 (1.01–1.17)	0.036
ΔDaytime SBP	1.06 (1.01–1.12)	0.028	1.07 (1.01–1.14)	0.031
ΔNighttime SBP	1.01 (0.96–1.06)	0.760	1.02 (0.97–1.08)	0.407
Δ24-h DBP	1.18 (1.05–1.33)	0.008	1.21 (1.05–1.40)	0.007
ΔDaytime DBP	1.13 (1.02–1.24)	0.015	1.16 (1.03–1.31)	0.014
ΔNighttime DBP	1.00 (0.94–1.07)	0.987	1.02 (0.95–1.10)	0.573
Δ24-h SBP load	1.03 (1.00–1.06)	0.087	1.05 (1.01–1.09)	0.010
ΔDaytime SBP load	1.02 (0.99–1.05)	0.134	1.04 (1.01–1.07)	0.022
ΔNighttime SBP load	1.02 (0.99–1.04)	0.167	1.03 (1.00–1.06)	0.045
Δ24-h DBP load	1.05 (1.01–1.09)	0.025	1.06 (1.01–1.11)	0.016
ΔDaytime DBP load	1.04 (1.01–1.09)	0.028	1.05 (1.01–1.10)	0.025
ΔNighttime DBP load	1.01 (0.99–1.03)	0.230	1.02 (1.00–1.04)	0.132
Δ24-h mean cSBPL	1.07 (1.01–1.13)	0.030	1.07 (1.01–1.14)	0.024
ΔDaytime mean cSBPL	1.06 (1.01–1.12)	0.017	1.08 (1.02–1.15)	0.010
ΔNighttime mean cSBPL	1.04 (0.95–1.13)	0.398	1.05 (0.96–1.15)	0.309
Δ24-h mean cDBPL	1.13 (1.01–1.26)	0.029	1.14 (1.01–1.29)	0.034
ΔDaytime mean cDBPL	1.12 (1.02–1.22)	0.016	1.14 (1.02–1.27)	0.017
ΔNighttime mean cDBPL	1.01 (0.90–1.13)	0.917	1.06 (0.93–1.21)	0.393
Δ24-h ARVs	1.07 (0.98–1.17)	0.138	1.09 (0.98–1.22)	0.103
ΔDaytime ARVs	1.05 (0.98–1.14)	0.173	1.07 (0.98–1.17)	0.140
ΔNighttime ARVs	1.03 (0.98–1.09)	0.278	1.04 (0.97–1.11)	0.286
Δ24-h ARVd	1.09 (0.99–1.20)	0.088	1.11 (0.99–1.24)	0.081
ΔDaytime ARVd	1.07 (0.99–1.16)	0.087	1.10 (1.00–1.21)	0.055
ΔNighttime ARVd	1.05 (0.98–1.12)	0.206	1.03 (0.96–1.11)	0.376

*HA, high altitude; AMS, acute mountain sickness; SBP, systolic blood pressure; DBP, diastolic blood pressure; cSBPL, cumulative systolic blood pressure load; cDBPL, cumulative diastolic blood pressure load; ARVs, average real variability of SBP; ARVd, average real variability of DBP; Δ, variation after HA exposure*.

### Correlation Between BP Parameters Variations and AMS

**Figure 4:**
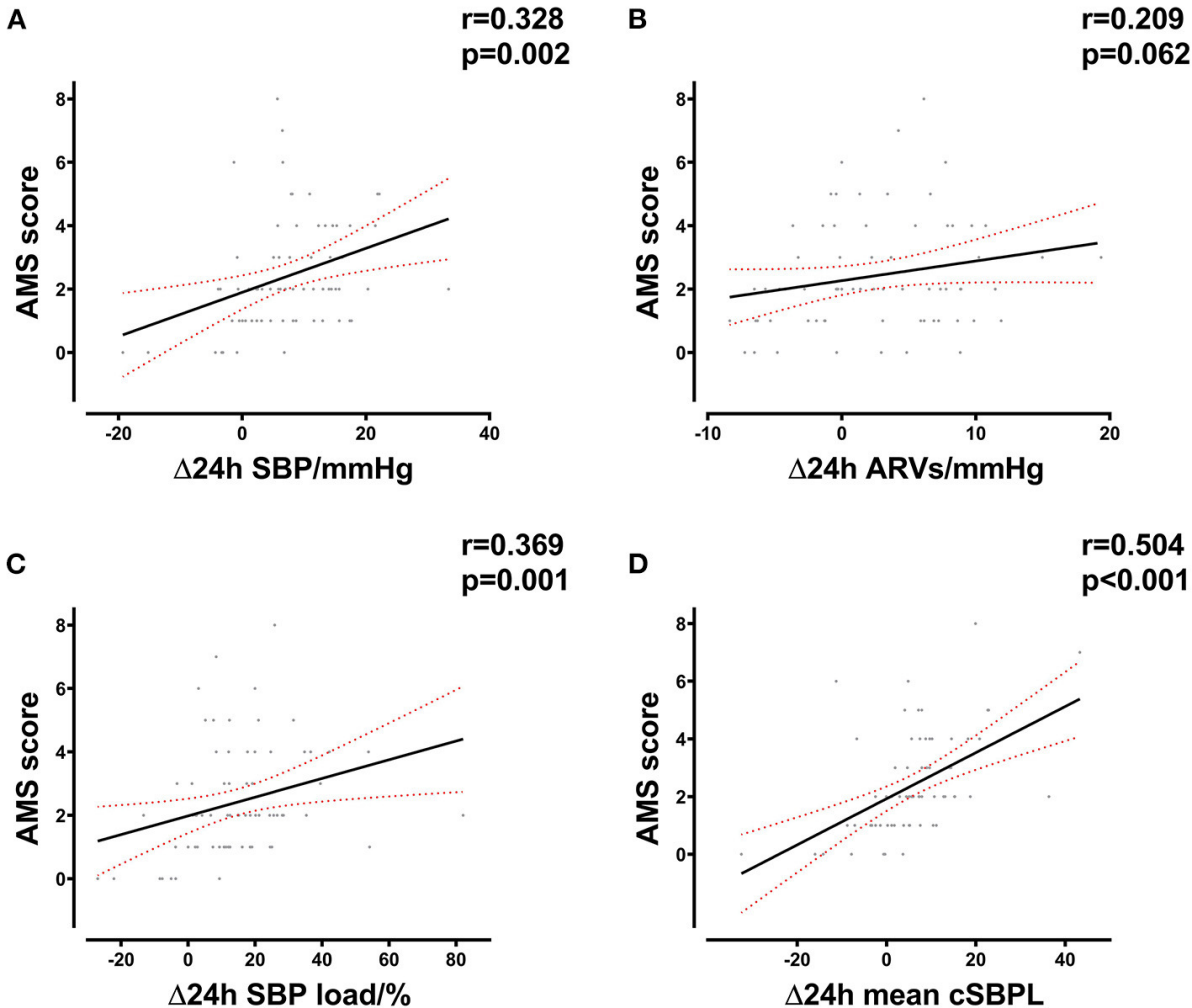
Correlation between delta 24-h SBP parameters and AMS score. **(A)** Correlation between delta 24-h SBP after HA exposure and AMS score in all subjects. **(B)** Correlation between delta 24-h ARVs after HA exposure and AMS score in all subjects. **(C)** Correlation between delta 24-h SBP load after HA exposure and AMS score in all subjects. **(D)** Correlation between delta mean 24-h cSBPL after HA exposure and AMS score in all subjects. HA, high altitude; AMS, acute mountain sickness; SBP, systolic blood pressure; cSBPL, cumulative systolic blood pressure load; ARVs, average real variability of SBP.

In order to more effectively evaluate the BP status of individuals and the relationship between BP changes and AMS during HA exposure, we analyzed the correlation between the changes in some BP indicators and the AMS. The variations in BP load (24-h SBP load variation, *R* = 0.369, *P* = 0.001; 24-h DBP load variation, *R* = 0.310, *P* = 0.005) as well as mean cBPL (24-h mean cSBPL variation, *R* = 0.504, *P* < 0.001; 24-h mean cDBPL variation, *R* = 0.290, *P* = 0.007) were also correlated with AMS score; which indicated that the BP load could also reflect BP status at HA and was associated with AMS (Figure 4; Supplementary Figure 1).

## Discussion

Our study is the first to demonstrate that changes in the BP load and AMS occur upon acute HA exposure in both healthy and AMS subjects. We analyzed the correlation between BP indicator variations and AMS. Interestingly, we observed that subjects with AMS displayed a higher BP level and BP load elevation than those without AMS after acute HA exposure. BP load changes during altitude may be closely correlated with the incidence of AMS. It could be an effective indicator of BP status assessment in AMS patients at HA.

### BP Changes After HA Exposure

BP remained largely unchanged over the first few minutes or hours after HA exposure. However, the BP increased remarkably and persisted with prolonged altitude exposure over the next few days. The rise appeared to be continuous and proportional to altitude. It was particularly pronounced at night, which resulted in a reduction in the decline in nocturnal BP; the circadian rhythm of 24-h BP fluctuations disappeared. BP primarily returned to the baseline level when on the return to sea level (18, 19). While in hypertensive subjects, a higher hypoxia-driven upward shift and steepening of the BP response to exercise were observed (20, 21). Initially, the secretion of vasodilator factors, such as nitric oxide, could counteract the effect of sympathetic activation (22). Subsequently, pressor mechanisms begin to dominate. The enhanced sympathetic nerve activity caused by hypobaric hypoxia is related to the sympathetic reflex stimulated by chemoreceptors in the carotid sinus and aortic arch, which stimulates the release of catecholamines. Mazzeo and Reeves have also confirmed this point that while the activity of the renin-angiotensin-aldosterone system was inhibited, the increase in vasoconstrictor factor secretion results in the elevation of peripheral vascular resistance caused by contraction of the smooth muscle arterioles (23). However, difference in peripheral α-adrenergic sensitivity to norepinephrine between sexes may produce different hypoxia-induced BP changes (24). Factors including endothelin-1 levels and erythropoietin are also potentially involved (25). Although cardiac diastolic relaxation was decreased, an elevated systolic function may result in BP increase (26).

### Clinical Meaning of BP Load

The BP load was introduced by pioneering studies published in the early 1990s (27). This may provide supplementary information for the diagnosis of hypertension in individuals when 24-h ABP would be misleading (27). At present, in addition to the mean BP, the degree of BP fluctuation has garnered increasing attention. Further, owing to the limited information provided by calculating the number of readings exceeding the normal BP threshold levels, several investigators have proposed a method for calculating the area under the curve (28). This reflects the extent and duration of the cumulative rise in BP, and may provide information that more closely reflects the actual BP status. In addition, other indicators that assess BP fluctuations, such as ARV, exhibited little independent prognostic significance (29). Previous studies suggested that the BP load was also closely associated with signs of target organ damage and adverse clinical cardiovascular events as the BP level (30, 31). Meanwhile, some studies have recommended to combining BP level and BP load to define hypertension and assess BP status (32). As no additional predictive value for the risk of target organ damage or cardiovascular complications after average the BP is considered (9, 33). However, the application of BP load to demonstrate BP status remains to be confirmed in extreme environments, and it may possess unique significance for the prediction and evaluation of HA cardiovascular disease.

### Association Between BP, BP Load and AMS

The relationship between changes in BP after altitude exposure and AMS remains controversial in previous studies. Although study has shown that BP remains relatively safe and stable at altitudes without to be symptomatic (34), it was also previously demonstrated that the BP elevation in AMS is relatively obvious after the acute plateau (35), and that higher BP at HA is related to the onset of AMS (7). Our previous study also revealed that the increase in BP during a certain period is related to sympathetic hyperexcitation, and excessive morning BP surge is associated with the onset of AMS (36). However, in some large size studies, increased BP after acute HA exposure has not been confirmed to be associated with AMS (37), and even a medical history of hypertension may be associated with a lower risk of AMS (37, 38). These conflicting results may be mainly due to individual differences among the populations included in the studies. For example, increased arterial thickness, decreased production of the vasodilator nitric oxide cerebral and lower blood flow might enable hypertensive patients less susceptible to AMS (15, 18). Thus, we excluded individuals with potential hypertension in this cohort study, which may have affected our observed results at HA in order to accurately reflect the relationship between BP changes during HA and the incidence of AMS. Because single BP measurement is not a good reflection of systemic circulation status, we also performed ABP measurement. Previous studies have confirmed some AMS risk factors. And certain factor, such as sex difference, may have an effect on BP changes during hypoxia (39, 40). We performed multivariate logistic regression and found BP load variation was independent risk factor of AMS after adjusting. Besides, we also demonstrated that there was a linear relationship between BP level variation and AMS although the degree of correlation was weak. Few previous reports have analyzed the relationship between high BP load and the risk of cardiovascular disease at HA. The excessive BP load is primarily due to the sympathetic excitation of the body (21). Moreover, AMS was associated with autonomic nervous dysfunction in previous studies. Patients with AMS development may accompanied with a disturbed BP control and an insufficient vasoconstriction function during altitude exposure (41, 42). And hyperactivation of the short-sympathetic after the acute plateau may be the main mechanism of pathogenesis. AMS may be induced by the elevation of BP after HA exposure, because an excessive rise in BP might result in symptoms, such as dizziness and headaches (36, 43). Therefore, we believe that BP load might be a good indicator of systemic circulation status assessment potentially during HA exposure. Our results also confirmed that changes in the BP load calculated using different methods are associated with AMS. Especially 24-h mean cSBPL showed a better correlation with AMS score compared with other ABP parameters. However, we also found that the variation in BP variability, which also reflects the degree of sympathetic excitation, was poorly associated with AMS. BP load may be a better indicator that reflect systemic circulation status in individuals with AMS at HA. A larger population study is required to confirm this point in the future. Notably, the explicit pathophysiology of AMS still remains unclear now. Previous study has proven that hypoxia could induce the blood-brain barrier disruption and lead to the development of cerebral edema subsequently. An increased brain volume with hypobaric hypoxia elevated intracranial pressure and impaired intracranial buffering capacity, which contributes to the development of the symptoms that define AMS (44). Sympathetic excitation causes the increase of systemic circulation BP load, cerebrovascular may also appear similar hemodynamic changes related to cerebral edema. But unfortunately, the relevant data were not tested. But at least, clinical evidences of the autonomic nervous system effects on AMS development could stimulate future research.

### Limitations

There are several limitations to the present study. Most importantly, due to the limitations of the HA conditions in this study, the BP data were not as complete as those of some studies conducted at LA, although the ABP recordings were still more than 80% of the total ABP data recorded. Therefore, we adjusted the calculation method of the cBPL by calculating the mean cumulative BP load. Notably, there might be a superior method of calculating the area under the fitting curve that is above the hypertension threshold (30). However, we did not use this method, because we were constrained by the discontinuity of the data. Secondly, the diagnosis of AMS was based on self-report, which could have led to classification bias. Moreover, we did not assess sleep status. Sleep may be disturbed during night ABP measurement. In addition, we did not examine some important indexes which could reflect the potential mechanism of AMS, such as cerebral hemodynamic monitoring. Lastly, different demography, such as race, smoking and drinking history may affect the established results. This conclusion is still need to be examined.

## Conclusion

To date, little is known about the relationship between BP load and AMS. Our study demonstrated that individuals with AMS exhibited higher BP levels and BP load changes after exposure to altitude. Excessive BP load variations are associated with AMS. BP load could be an effective indicator of systemic circulation status in AMS patients. These results may provide novel insights into the mechanisms underlying the occurrence of AMS.

## Data Availability Statement

The original contributions presented in the study are included in the article/Supplementary Material, further inquiries can be directed to the corresponding author/s.

## Ethics Statement

The studies involving human participants were reviewed and approved by Human Ethics Committee of the Xinqiao Hospital, Third Military Medical University (Identification code, 201907501) which registered at www.chictr.org.cn (ChiCTR-TRC-No.1900025728). The patients/participants provided their written informed consent to participate in this study. Written informed consent was obtained from the individual(s) for the publication of any potentially identifiable images or data included in this article.

## Author Contributions

RChen and LH worked on the conception of the study. RChen, MS, YY, JK, FY, CH, RCheng, and HL contributed to the data collection. CL and JY checked the data. RChen and XY performed the statistical analysis. RChen drafted the manuscript. XG, CL, JY, HT, JZ, and LH reviewed the manuscript. All authors read and approved the final version of the manuscript.

## Funding

This work was supported by grants from the National Natural Science Foundation of China (Grant No. 81730054), Research Project of PLA (Grant No. BLJ18J007), and the Ministry of Health of P.R. China (Grant No. 201002012).

## Conflict of Interest

The authors declare that the research was conducted in the absence of any commercial or financial relationships that could be construed as a potential conflict of interest.

## Publisher's Note

All claims expressed in this article are solely those of the authors and do not necessarily represent those of their affiliated organizations, or those of the publisher, the editors and the reviewers. Any product that may be evaluated in this article, or claim that may be made by its manufacturer, is not guaranteed or endorsed by the publisher.

## Supplementary Material

Supplementary Figure 1 Correlation between delta 24-h DBP parameters and AMS score. **(A)** Correlation between delta 24-h DBP after HA exposure and AMS score in all subjects. **(B)** Correlation between delta 24-h ARVd after HA exposure and AMS score in all subjects. **(C)** Correlation between delta 24-h DBP load after HA exposure and AMS score in all subjects. **(D)** Correlation between delta mean 24-h cDBPL after HA exposure and AMS score in all subjects. HA, high altitude; AMS, acute mountain sickness; DBP, diastolic blood pressure; cDBPL, cumulative diastolic blood pressure load; ARVd, average real variability of DBP.
