# Supplementary figures and images for: Blood Pressure Load: An Effective Indicator of Systemic Circulation Status in Individuals With Acute Altitude Sickness
Source: Front Cardiovasc Med. 2022 Jan 3;8:765422. doi: 10.3389/fcvm.2021.765422 (PMC8761955; doi:10.3389/fcvm.2021.765422)

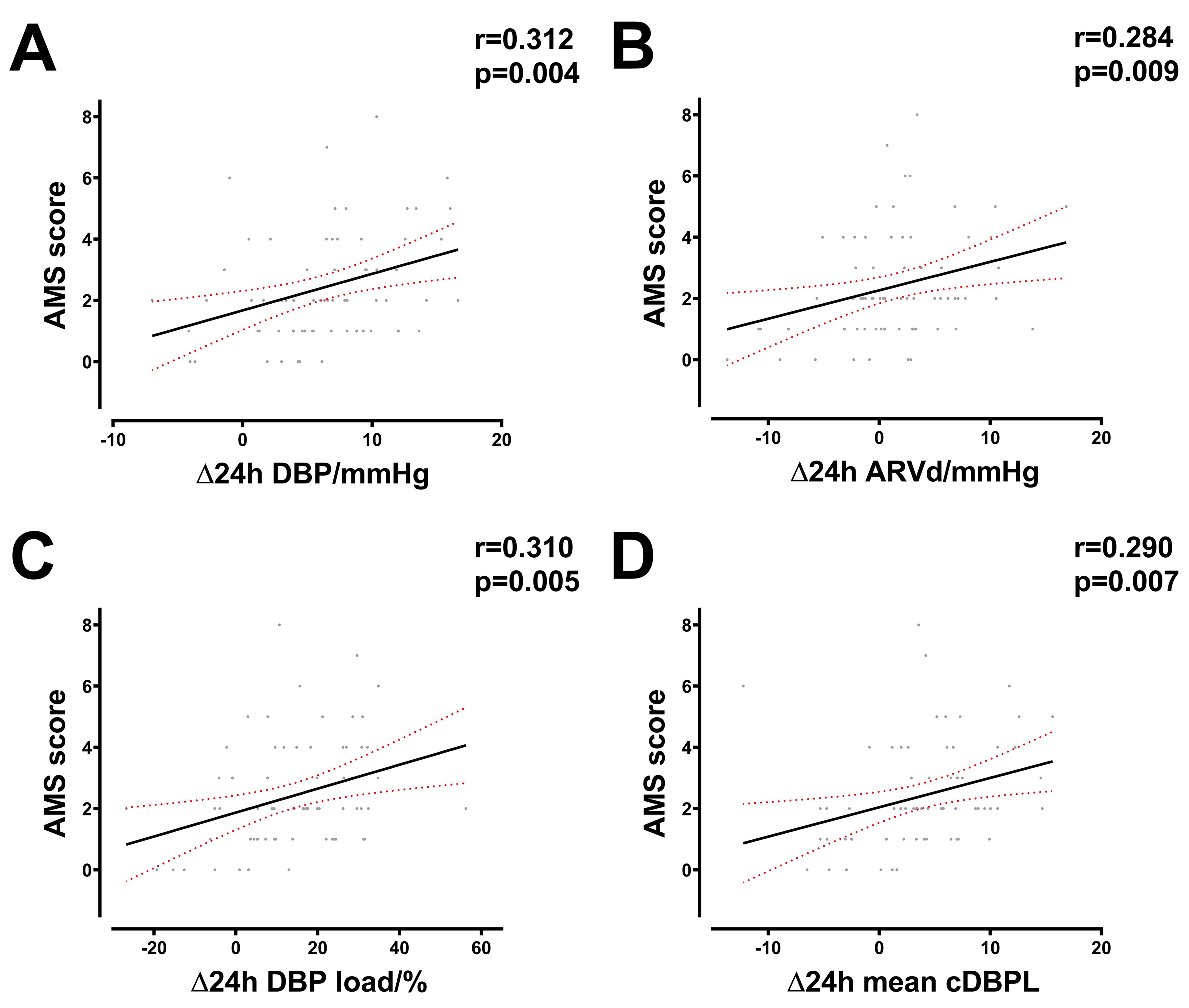

Supplement: Supplementary Figure 1 — Correlation between delta 24-h DBP parameters and AMS score. (A) Correlation between delta 24-h DBP after HA exposure and AMS score in all subjects. (B) Correlation between delta 24-h ARVd after HA exposure and AMS score in all subjects. (C) Correlation between delta 24-h DBP load after HA exposure and AMS score in all subjects. (D) Correlation between delta mean 24-h cDBPL after HA exposure and AMS score in all subjects. HA, high altitude; AMS, acute mountain sickness; DBP, diastolic blood pressure; cDBPL, cumulative diastolic blood pressure load; ARVd, average real variability of DBP. [file Image_1.TIF]
